# Nanocellulose Membranes for Plasmon-Enhanced Removal of Organic Pollutants from Water

**DOI:** 10.1021/acsanm.5c04857

**Published:** 2026-01-03

**Authors:** Sivoney Ferreira de Souza, Christina Beresowski, Sabine Kosmella, João Ameixa, Bhanu Kiran Pothineni, Adrian Keller, Matthias Hartlieb, Andreas Taubert, Ilko Bald

**Affiliations:** † Institute of Chemistry, 26583 University of Potsdam, Karl-Liebknecht-Str. 24-25, 14476 Potsdam, Germany; ‡ Centre of Physics and Technological Research (CEFITEC), Department of Physics, NOVA School of Science and Technology, University NOVA of Lisbon, Campus de Caparica, 2829-516 Lisbon, Portugal; § Technical and Macromolecular Chemistry, Paderborn University, Warburger Str. 100, 33098 Paderborn, Germany; ∥ Fraunhofer Institute for Applied Polymer Research (IAP), Geiselbergstraße 69, 14476 Potsdam, Germany

**Keywords:** organic pollutants, water treatment, plasmon resonance, cellulose nanofibers, AgNP in situ synthesis, vancomycin

## Abstract

We present multifunctional cellulose nanofiber (CNF) membranes containing in situ-synthesized plasmonic silver nanoparticles (AgNPs) for the efficient removal of organic pollutants from water. These membranes combine the adsorptivity and mechanical strength of CNF with the plasmonic properties of AgNPs, which, when activated by blue light, enable the photocatalytic degradation of organic contaminants such as dyes and pharmaceuticals and thereby enhance their removal from water. The membranes demonstrate excellent adsorption capacity, flexibility, mechanical durability, and reusability and show no AgNP leaching in water, making them ideal for sustainable water treatment applications. Performance tests show significantly higher pollutant removal rates in membranes containing AgNPs, and exposure to blue light further accelerates the removal process, ascribed to the plasmonic decomposition of pollutants at the surface of the CNF_AgNP composite. To validate these findings, we tested methylene blue and the antibiotic vancomycin. The results confirm the plasmonic degradation of the molecules by irradiation of the CNF_AgNP membranes with visible light. These membranes offer a promising solution for water purification due to enhanced adsorption combined with photodecomposition by visible light, advancing sustainable technologies for tackling water contamination.

## Graphical abstract

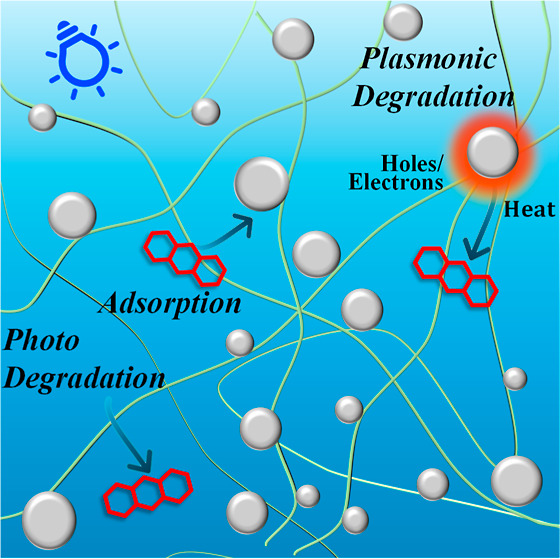

## 1 Introduction

Localized surface plasmon resonance (LSPR) in metallic nanoparticles arises from the coherent oscillation of conduction electrons when these particles are illuminated by light of specific wavelengths^1^ This phenomenon generates enhancements in local electromagnetic fields near the nanoparticle surface and optical absorption, particularly in the visible range, enabling a diverse range of applications, including chemical and biological sensing, surface-enhanced Raman scattering,^2,3^ and photocatalysis.^4^ The decay of LSPRs occurs through radiative and nonradiative pathways. Radiative decay results in the emission of photons, as the excited electrons return to their ground state, while nonradiative decay involves the transfer of nonthermal charges to the surrounding as well as energy dissipation as heat due to electron–phonon interactions within the metal lattice.^5^

Plasmonic catalysis uses plasmonic nanoparticles for driving chemical reactions under light illumination, mostly in the visible range. The LSPR excitation results in the generation of nonthermal charge carriers (electron/hole pairs) that can be transferred to nearby reactants, effectively lowering the activation energy required for chemical transformations and enabling reactions that are energetically unfavorable.^6–9^ While applications range from molecular hydrogen dissociation^10^ or generation,^11^ propylene epoxidation,^12,13^ ^,^ oxidation of carbon monoxide,^13^ and ammonia oxidation^13^ to the dimerization of 4-nitrothiophenol,^14,15^ their potential for environmental applications, such as wastewater treatment, remains to be explored.^16–21^

The increasing prevalence of organic pollutants in aquatic environments raises concerns regarding their impacts on human health and ecosystems.^22^ These persistent pollutants are frequently found in trace concentrations (<μg/L) and commonly referred to as micropollutants, which include pharmaceuticals and pesticides.^23^ They are frequently detected in surface waters and drinking water at concentrations ranging from ng·L^–1^ to low μg·L^–1^, while conventional wastewater treatment plants often achieve removal efficiencies below 50–70% for many persistent compounds.^22,23^

Human use of antibiotics is widespread, with models estimating that of ∼30,300 tons of the 40 most used antibiotics consumed annually, approximately 9500 tons (∼31%) are released into river systems, leading to antibiotic concentrations above ecotoxicological thresholds over an estimated 6 million kilometers of global rivers under low flow conditions.^24^ Measured antibiotic concentrations in surface waters can be substantial, with reported levels of up to 15 μg·L^–1^ in the Americas, over 10 μg·L^–1^ in Europe and Africa, and exceeding 450 μg·L^–1^ in some Asian-Pacific regions, concentrations that are high enough to affect microbial communities and potentially promote resistance.^25^

Their removal by conventional water treatment systems is highly inefficient, resulting in their accumulation in natural aquatic systems and even tap water.^26^ This accumulation contributes to ecological disturbance, bioaccumulation in food chains, and antimicrobial resistance, emphasizing the need for innovative remediation technologies.

Advanced oxidation processes are widely applied for micropollutant removal, relying on reactive oxidative species, primarily ^•^OH and SO_4_^•–^, to oxidize target contaminants.^27,28^ While processes like ozonation and UV-based methods (e.g., O_3_/UV and UV/H_2_O_2_) are well-established in water treatment, their high operational costs, energy-intensive artificial UV sources, and secondary waste generation present significant limitations^27^ These challenges underscore the need for alternative technologies capable of achieving efficient pollutant degradation under more sustainable conditions.

Plasmonic catalysis offers a transformative approach, utilizing sunlight or low-cost artificial light to activate hybrid nanostructures composed of plasmonic metals (e.g., Au and Ag) and semiconductors (e.g., TiO_2_, ZnO, and SiO_2_).^17^ These plasmonic nanoparticle-based heterojunction photocatalysts enhance pollutant degradation through mechanisms such as hot carrier generation, charge separation, energy transfer, heat, and electromagnetic field enhancement. Despite their promise, the integration of plasmonic materials into scalable and cost-effective platforms for water treatment is yet to be fully realized.^17,29,30^ The semiconductor titanium dioxide (TiO_2_) was used in combination with AgNPs for the degradation of the dyes rhodamine B and methyl orange after 3 h exposure to a solar simulator.^20^ In another study, thin TiO_2_ films were combined with metallic plasmonic nanostructures of aluminum and gold that increased the optical absorption of the TiO_2_ films to above 70% in the visible and NIR spectral range.^19^ This enhanced the photocatalytic activity for the degradation of organic dyes and the pharmaceutical drug carbamazepine under different light sources. The concentration of the latter was reduced to 48% in 360 min. Graphene-based nanomaterials have been widely explored for environmental remediation owing to their high surface area and tunable surface chemistry, enabling efficient adsorption and catalytic removal of organic pollutants.^31^ These studies emphasize the effectiveness of nanostructured composite membranes as functional platforms for pollutant capture and transformation, providing a conceptual basis for developing nanocellulose-based membranes incorporating plasmonic nanoparticles for light-assisted remediation processes.

In yet another work, cotton nanocomposite fibers containing Au/Ag have been produced for efficient solar water desalination and wastewater treatment,^18,32^ and plasmonic nanoparticle-based heterojunction photocatalysts, like Janus gold nanorods partially coated with silica, have explored to activate persulfate (PDS) to degrade organic pollutants under more extreme conditions (e.g., 48 °C, four simulated suns, and the presence of oxidative agents). Similarly, Miralles et al. have demonstrated the use of citrate-stabilized AgNPs and AuNPs for the oxidation of arsenic, converting toxic arsenite As­(III) into less harmful arsenate As­(V).^33^ They achieved complete arsenic conversion under visible light and an excess of H_2_O_2_.

Herein, we present a novel catalytic platform comprising silver nanoparticles (AgNPs) embedded within a cellulose nanofiber (CNF) membrane, termed CNF_AgNP, for the removal of organic pollutants under blue light irradiation. CNFs, derived from cellulose, offer high surface area, mechanical strength, certain transparency, and biodegradability, making them ideal scaffolds for nanoparticle immobilization.^34,35^ In the context of photo regeneration processes for cofactor molecules (NADH, nicotinamide adenine dinucleotide) using plasmonic materials,^36^ use of cellulose as a biocompatible and inert scaffold to support plasmonic nanoparticles was proposed, specifically bimetallic palladium-coated gold nanorods (AuPdNRs). They demonstrated that the combination of AuPdNRs with cellulose in a photocatalytic composite increases the photoregeneration rate of NADH compared to systems without the scaffold under visible light illumination. Overall, unlike traditional supports requiring ligands or capping agents (e.g., CTAB and citrate), cellulose facilitates nanoparticle stabilization, preventing uncontrolled aggregation on solid substrates without compromising surface accessibility or photochemical activity.^36,37^ Also, nanocellulose in general has high adsorptive capacity, and filters are a common and attractive sustainable way to treat wastewater, as demonstrated for the removal of crystal violet,^38^ proteins,^39^ and metal ions.^40,41^ Recent advances in covalent organic framework–based membranes have shown how highly ordered porous polymer networks can be engineered into mechanically stable membrane architectures, enabling efficient and reusable platforms for the removal of organic pollutants and metal ions from water.^41^ Similar to this membrane-based design strategy, nanocellulose membranes incorporating plasmonic nanoparticles offer an alternative and sustainable route, where a robust fibrillar network stabilizes light-responsive metal nanostructures to promote pollutant adsorption and light-driven transformation processes. Overall, research into plasmonic reactions using sustainable materials has shown significant potential for water contamination treatment.^42,43^

We hypothesize that blue light irradiation of CNF_AgNP membranes will drive the degradation of organic pollutants through synergistic adsorption of molecules to the CNF and light-induced electron and heat transfer mechanisms requiring less stringent conditions compared with previous studies. Using methylene blue (MB) and vancomycin (Van) as models for water contaminants, we demonstrate pollutant removal kinetics and highlight the complementary roles of adsorption and plasmonic photocatalysis. This study provides a proof of concept for a scalable plasmonic nanoparticle-enabled water treatment technology, paving the way for sustainable nanophotonic solutions for environmental remediation.

## 2 Experimental Section

### 2.1 Membrane Production

The commercial CNF was first dispersed in Milli-Q water at a concentration of 1% (w/w). The CNF suspension was then passed once through an M-110P Microfluidizer (Microfluidics) at a pressure of 1000 bar. An exception occurred for the CNF–AgNP membranes used in the reactions under green, red, and white light (Figure 4). For these experiments, the corresponding CNF suspension underwent three homogenization cycles at 700 bar, as this new batch of CNF required different processing conditions to be successfully formed into membranes. To prepare the CNF membranes, the CNF suspension was mixed with glycerol to achieve a final concentration of 1% (w/w). This mixture was subjected to a solution-casting process in Petri dishes (90 mm in diameter) and placed in an oven at 45 °C for 48 h to form the membranes.^44^

The in situ synthesis of AgNPs on the CNF matrix was carried out using a modified procedure of silver mirror reaction.^45^ First, 50 mL of a 1.5% AgNO_3_ solution was mixed with 2.5 mL of a 2.5% NaOH solution in an ice bath. A 30% NH_3_ solution was added dropwise until the brownish residue was completely dissolved. This solution was then added to the CNF membranes, and the membranes were allowed to react for approximately 6 min. After the reaction, the membranes were removed, washed thoroughly with water, and dried in the oven at 40 °C for 24 h.

#### 2.1.1 Atomic Force Microscopy

The morphology and dimensions of the CNFs were characterized by using atomic force microscopy (AFM). CNF suspensions (5 mg/L) were prepared and deposited on a mica surface. AFM imaging was conducted using a Bruker Multimode 8 AFM in tapping mode, employing a Nanoworld cantilever with a stiffness of 42 N/m and a resonance frequency in the range of 260–32 kHz. The images obtained were processed and analyzed by using Gwyddion software. A total of 100 individual nanofibers were measured to determine the average dimensions.

#### 2.1.2 Scanning Electron Microscopy

Scanning electron microscopy (SEM) was used to examine the surface morphology of the CNF–AgNP membrane after gold sputtering (5 nm). The imaging was performed by using a ZEISS UltrPlus SEM, utilizing secondary electron detection.

#### 2.1.3 Transmission Electron Microscopy

For transmission electron microscopy (TEM) analysis, a 1 cm^2^ piece of the CNF_AgNP membrane was cut into small sections and immersed in 10 mL of deionized water. The sample was kept in bath sonication for 60 min. After the mechanical treatment, a droplet of the suspension was collected, deposited onto a TEM sample holder, and dried for 24 h. TEM imaging was carried out using a JEOL JEM 1011.

#### 2.1.4 High Resolution-Transmission Electron Microscopy

The same sample preparation from TEM was performed here. TEM imaging was carried out using a JEOL (Jeol 2200FS) using 200 kV as the acceleration voltage. The diameters of the AgNP were measured on the high resolution transmission electron microscopy (HR-TEM) images using ImageJ 1.54g software.

#### 2.1.5 Energy-Dispersive X-ray Spectroscopy

Energy-dispersive X-ray spectroscopy (EDX)-TEM was performed on the same HR-TEM sample and with the same equipment of HR-TEM The EDX detector is also from Jeol equipment. Several spectra were taken, and one spectrum is shown with its related particle, which was measured.

#### 2.1.6 X-ray Diffraction

X-ray diffraction (XRD) of the membranes of CNF and CNF_AgNP was carried out in the X-ray equipment, Empyrean diffractometer (Malvern Panalytical, Netherlands) equipped with a Cu X-ray source. The samples were placed between Kapton films before being inserted in the ogoliometro. Later, the XRD spectra were collected in the step of 0.2° for the angles of 2θ between 4 and 70°.

#### 2.1.7 Zeta Potential

The ζ-potential (electrophoretic mobility) of the CNF suspensions was measured using a ZetaSizer Nano SZ4 3000 at 25 °C. The CNF concentration in water was 0.001 wt %. Three separate measurements were performed for each sample to ensure the accuracy.

#### 2.1.8 Fourier Transform Infrared Spectroscopy

Fourier transform infrared (FTIR) spectra were obtained using a Thermo Scientific Nicolet iS instrument equipped with a Thermo Scientific iD7 ATR accessory. A total of 32 scans were collected for each measurement.

#### 2.1.9 Rheology

Rheological measurements were performed by using a stress-controlled rheometer (Gemini 150) from Bohlin. Oscillatory shear measurements were conducted using a 40 mm plate–plate geometry in the frequency range of 0.01 to 5 Hz.

#### 2.1.10 Absorbance Spectrum

To obtain the absorbance spectrum of the CNF–AgNP membrane, it was measured the reflectance (*R*) and transmittance (*T*) using the UV–vis spectrometer. The absorbance (*A*) was then calculated using the relation

A(%)=100−R−T

where *R* (%) and *T* (%) correspond to the measured reflectance and transmittance, respectively.

### 2.2 Pollutant Removal Tests

The Nano cube photoreactor from Thalesnano was employed to monitor all reactions. A bath cooling system was integrated in the equipment to keep the temperature of the photoreactor at around 24 °C. Four LEDs with a power of 128 W generating blue (457 nm), green (523 nm), red (623 nm), and white light were used for irradation, while stirring the reaction volume with a magnetic stirrer at 270 rpm. For each reaction, 1 cm^2^ of the CNF_AgNP membrane was used for every 1 mL of solution containing the target molecule to be degraded. The CNF and CNF_AgNP membranes were cut to a size of approximately 1 cm × 4 cm; since the density of material had some variability, the mass was also measured and kept constant at around 27.00 ± 0.13 mg, and 4 mL of solution was tested. The glass flask (4 mL) was used and kept closed during the reactions, containing the magnetic bar inside. For the dark reactions, the flasks were covered with aluminum foil to avoid a light effect.

#### 2.2.1 Methylene Blue Dye

To investigate the plasmonic activity of the CNF_AgNP membranes in degrading MB, an aqueous solution of MB (0.02 mM) was prepared. The degradation process was monitored using a UV–visible absorption spectrophotometer (UV-2600 Shimadzu) at 5 min intervals following the peak at 660 nm. Identical experiments were conducted under dark conditions and with a CNF membrane, keeping the same ratio of membrane/solution and quantity of the membrane.

#### 2.2.2 Vancomycin

A solution with a concentration of 0.25 mM was used to study Van decomposition by the plasmonic reaction in the CNF_AgNP membrane under illumination. The degradation was tracked using a UV–visible absorption spectrophotometer (UV-2600 Shimadzu) every 5 min following the peak of 280 nm. Identical experiments were conducted under dark conditions and with a CNF membrane, with the same Van concentration and the same ratio of membrane/solution as mentioned before.

#### 2.2.3 Repetitions of Reactions

In order to distinguish more clearly the plasmonic degradation from the adsorption effect, the reaction was performed five times using the same CNF_AgNP membrane with irradiation and in dark conditions inside the photoreactor at the same conditions as described previously for MB and Van solution. This experiment was done two times, and the average and error bars are shown for the 5th reaction.

#### 2.2.4 Kinetic Analysis

The kinetics of the MB dye and Van removal were analyzed by using the first-order kinetic model. The pollutant removal was monitored by measuring the absorbance of the solution at specific time intervals using a UV–visible absorption spectrophotometer with a 10 cm path-length quartz cuvette. For first-order kinetics, the removal of the pollutants followed the equation

$$\ln\left(\frac{A}{A_{0}}\right)=-k\cdot t$$

where *A* and *A*_0_ are, respectively, the concentration of the pollutant at time *t* and at the start of the reaction, *k* is the apparent first-order rate constant (min^–1^), and *t* is the time in minutes. A linear regression was used to obtain the value of the rate constant (*k*) from the slope of the plot of ln­(*A*/*A*_0_) versus time. For each pollutant, the kinetics of the apparent first-order kinetic constants were calculated from the slope of the linearized plot.

#### 2.2.5 Adsorption Capacity

A Van solution with an initial concentration of 0.25 mM was prepared, and each CNF–AgNP membrane sample (1 cm^2^) was immersed in 100 mL of this solution in triplicate. The adsorption capacity *q*_e_ (mg·g^–1^) of the CNF–AgNP membrane was evaluated using Van as a model molecule. After the adsorption period, the remaining concentration of Van in the solution was determined by UV–Vis absorption spectroscopy using a previously established calibration curve. The adsorption capacity was then calculated according to

$$q_{e}=\frac{\left(C_{0}-C_{e}\right){\cdot V\cdot M}_{MW}}{m}$$

where: *q*_e_—the adsorption capacity (mg·g^–1^), *C*_0_—initial concentration (0.25 mmol/L), *C*_e_ = equilibrium concentration (mmol/L), *M*_MW_—molar mass (g/mol) of Van, *m*—mass of dried membrane (g), *V*—volume Van solution (l).

Adsorption equilibrium was confirmed by maintaining the membrane in contact with the solution for approximately 1 week, during which no further variation in concentration was detected. The amount of adsorbed Van was further evaluated by measuring the mass increase of the membrane after drying at 45 °C for 24 h.

### 2.3 Temperature Behavior

The temperature acquisition was monitored after the use of the Photocube photoreactor from ThalesNano, as described before, with 4 LEDs or 100% power of blue light irradiation (wavelength of 457 nm). For that, the same conditions of the reactions were kept at 4 mL of water to 27 mg of membrane. So, one glass flask was kept with just water; another with the CNF membrane and water; and another with CNF_AgNP and water. The temperature was measured using a thermopar for 0, 5, 10, 15, 20, 30, and 60 min under irradiation.

### 2.4 Bacterial Inhibition Assay

To test the in vitro susceptibility of bacteria to both active and inactive Vans, a bacterial inhibition assay was performed. *Bacillus subtilis* was cultured in LB medium at 37 °C with constant shaking overnight. The following day, 200 μL of overnight-grown bacteria was transferred to 35 mL of fresh medium and allowed to grow until the optical density (OD) at 600 nm reached 0.3. The inhibition assay was conducted using the broth microdilution method with Van concentrations of 3 and 2 μg/mL in a 96-well plate, performed in triplicate. The OD at 600 nm was measured at 0 and 24 h using a microplate reader (Berthold Tristar^2^ S, Germany).

## 3 Results

### 3.1 CNF_AgNP Membrane Production and Characterization

The synthesis procedure for the CNF_AgNP membranes is schematically illustrated in Figure 1a, with detailed methods provided in the Supporting Information. In the first step, CNFs were dispersed in water using a microfluidizer and cast into membranes in Petri dishes, followed by a drying process. Subsequently, AgNPs were synthesized in situ on the pure CNF membranes via a modified silver mirror reaction,^45^ as described in Supporting Information, followed by a drying process. This resulted in robust and flexible CNF_AgNP membranes, as shown in Figure 1a, where digital images highlight a distinct color change from translucid (pure CNF) to brownish (CNF_AgNP), indicating the presence of AgNPs. The translucidity of the pure CNF membrane enhances the efficient exposure of AgNPs to an external light source.

**Figure 1:**
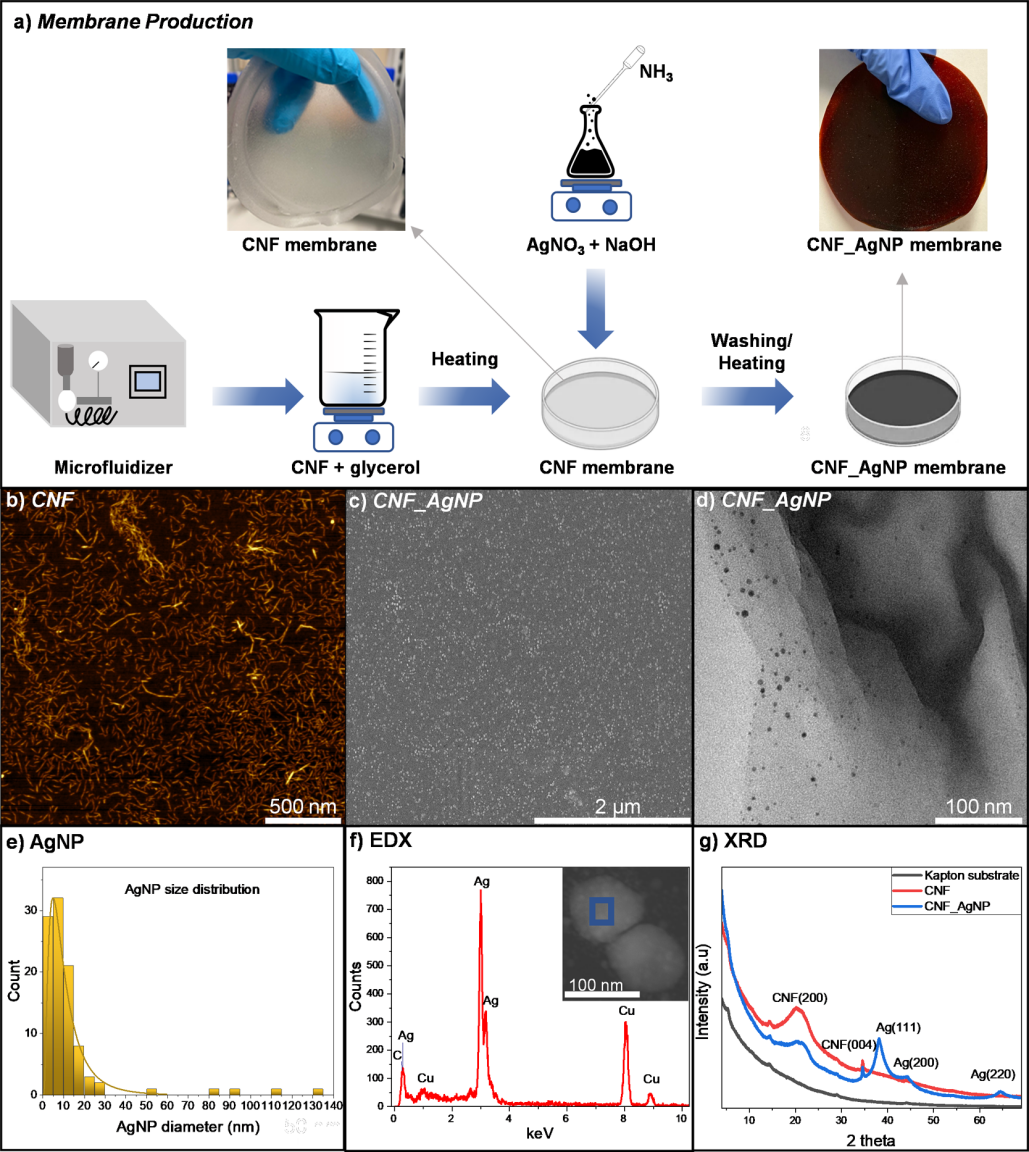
Production and characterization of CNF_AgNP membranes: (a) schematic illustration of the CNF and CNF_AgNP membrane production process, along with photographs comparing pure CNF and CNF_AgNP membranes. (b) AFM images showing the nanoscale topography of CNFs. (c) SEM images showing the surface morphology of a CNF_AgNP membrane, highlighting the distribution of AgNPs. (d) TEM images showing the dispersion of AgNPs within the CNF matrix after sonication, demonstrating their stability. (e) AgNP size distribution counted by ImageJ software from HR-TEM images shown in the Supporting Information (Figure S1). (f) EDX spectrum measured on the AgNP presented in the HR-TEM image. (g) XRD results from the CNF and CNF_AgNP membrane elucidating the main peaks of their crystalline planes.

AFM was employed to characterize the morphologies of CNFs. The microfluidization process enhanced the dispersion of CNF and facilitated the breakdown of macrofibers and microfibers into individual nanofibers. AFM images (Figure 1b) reveal well-isolated nanofibers with an average diameter of 15.7 ± 4.3 nm and length of 117.8 ± 70.8 nm. Some nanofibers can appear entangled and folded, forming an interconnected and interlocked network, which is beneficial for membrane production, as it provides structural integrity.

SEM images (Figure 1c) confirm the homogeneous distribution of AgNPs on the membrane surface, while TEM images (Figure 1d) reveal well-dispersed AgNPs embedded within the CNF matrix. To further evaluate the AgNPs, TEM analysis was performed after subjecting small pieces of the CNF_AgNP membrane to mechanical stirring and bath sonication for 30 min per day during 10 consecutive days. The TEM image (Figure 1d) shows a fragment of the CNF membrane containing dispersed AgNPs, confirming their uniform incorporation. This morphological characterization shows that AgNPs are compactly embedded within the CNF matrix, ensuring strong mechanical integrity and durability during photocatalytic applications. Figure 1e shows the size distribution of AgNP diameters based on HR-TEM images presented in the Supporting Information (Figure S1). Fitting of the size distribution with a log–normal model reveals that 95% of the AgNPs have sizes below 17.6 nm, with a maximum at 4 nm.

EDX was performed, and the analysis confirmed the presence of Ag, as well as C and Cu originating from the TEM grid. The XRD patterns (Figure 1f) show diffraction patterns corresponding to the crystalline planes characteristic of cellulose (200, 004) and to metallic silver (111, 200, 220), indicating the formation of AgNPs. Similar patterns were also reported by Shin et al., who reduced AgNPs on the CNF surface and observed that TEMPO-modified CNF promoted increased content of AgNP formation due to the presence of additional carboxylic groups compared with unmodified CNF.^46^ The XRD measurements were performed with Kapton films, giving rise to the background signal observed in Figure 1g. Additional AFM imaging of the CNF_AgNP membrane (Figure S2) further confirms the fibrillar organization of the nanocellulose network within the composite. While certain surface features suggest the presence of AgNPs, individual nanoparticles cannot be unambiguously resolved. This is attributed to the nonplanar topography of the membrane and the very small size of the AgNPs (≈5 nm), whose height contribution is comparable to the intrinsic nanoscale variations of the CNF matrix. Consequently, AFM provides reliable visualization of the CNF network but limited contrast for resolving discrete AgNPs embedded within the composite structure.

The mechanical properties of the CNF_AgNP membranes were assessed through rheological measurements, revealing solid-like behavior for both pure CNF and CNF_AgNP membranes; detailed rheological data are presented in the Supporting Information (Figure S3). The storage modulus (*G*′) was consistently higher than the loss modulus (*G*″), indicating a predominant gel response. The storage modulus of the CNF membrane increases over a certain frequency, suggesting an increase in strong interactions among the CNF network. Additionally, the high aspect ratio of CNF likely contributes to the enhancement in yield stress, promoting fiber entanglement.^47^ The presence of AgNPs slightly decreases membrane viscosity, but both materials exhibit similar trends, with viscosity decreasing at higher frequencies. This suggests that AgNPs are well-integrated within the CNF matrix without altering the overall viscosity behavior. The increase in the storage modulus (*G*′) further suggests enhanced nanoparticle interactions, leading to higher stiffness and compactness. Additionally, during water exposure experiments, the CNF_AgNP membranes demonstrated enhanced mechanical robustness, whereas pure CNF membranes presented swelling caused by their higher water affinity. This highlights the superior stability of the CNF_AgNP membranes, making them suitable for water treatment applications.

FTIR spectroscopy and thermogravimetric analysis (TGA) further confirmed the successful incorporation of AgNPs into the CNF matrix. FTIR (Figure S4a) spectra indicated the introduction of carboxylate groups (COO^–^) and other oxygen-containing functionalities, suggesting partial oxidation of cellulose during AgNP synthesis. The carboxylate group can attach silver ions to the cellulose, providing a site for their growth into AgNPs. Further, the carboxylate groups can stabilize the particles electrostatically.^48^ CNF is known to exhibit strong electrostatic interactions depending on the functional groups present on its surface that can interact with surrounding molecules, leading to charge-dependent repulsion or attraction effects.^49,50^ To further investigate this, the zeta potential of the CNF suspension was measured, yielding an average value of −60 ± 15 mV.

TGA (Figure S4b) demonstrated that CNF_AgNP membranes exhibit similar degradation temperatures compared to pure CNF membranes, with the main degradation peak occurring at around 300 °C (as seen in the derivative thermogravimetry curve). However, after 600 °C, an additional 23% residual mass was observed in the CNF_AgNP membranes, attributed to the presence of AgNPs. Detailed thermal analysis spectra are provided in the Supporting Information.

### 3.2 Performance of Plasmonic CNF_AgNP Membranes in Organic Pollutant Removal from Water: the Role of the AgNP/CNF/Water Interface

The performance of CNF_AgNP membranes in removing organic pollutants from water was investigated through independent studies on representative pollutants: MB, a synthetic dye, and Van, a widely used antibiotic. These studies were designed to evaluate both adsorption and plasmonic degradation mechanisms, considering the distinct chemical properties and environmental relevance of each pollutant.

#### 3.2.1 Methylene Blue Degradation: Photocatalytic Enhancement by Plasmonic AgNPs

The removal of 0.02 mM MB from an aqueous solution was assessed by monitoring the absorbance of MB over time via UV–vis spectrophotometry. 4 mL of the solution was kept in contact with 27 mg of the membranes, corresponding to approximately 4 cm^2^ for CNF_AgNP and 2 cm^2^ for CNF. Studies of the adsorption of MB to the membranes were conducted under dark and light conditions, the latter using blue irradiation (λ = 453 nm). Detailed experimental methods and a description of the photoreactor are available in Supporting Information.

The UV–vis spectra in Figure 2a indicate significant MB removal when CNF_AgNP membranes were used, as indicated by the decrease in absorbance at 664 nm.^51^ Figure 2b compares MB removal kinetics (*A*/*A*_0_ vs time) across different conditions: photodegradation (blue light alone), adsorption under dark conditions (CNF, CNF_AgNP), and combined removal by adsorption-photodegradation (CNF_AgNP under illumination).

**Figure 2:**
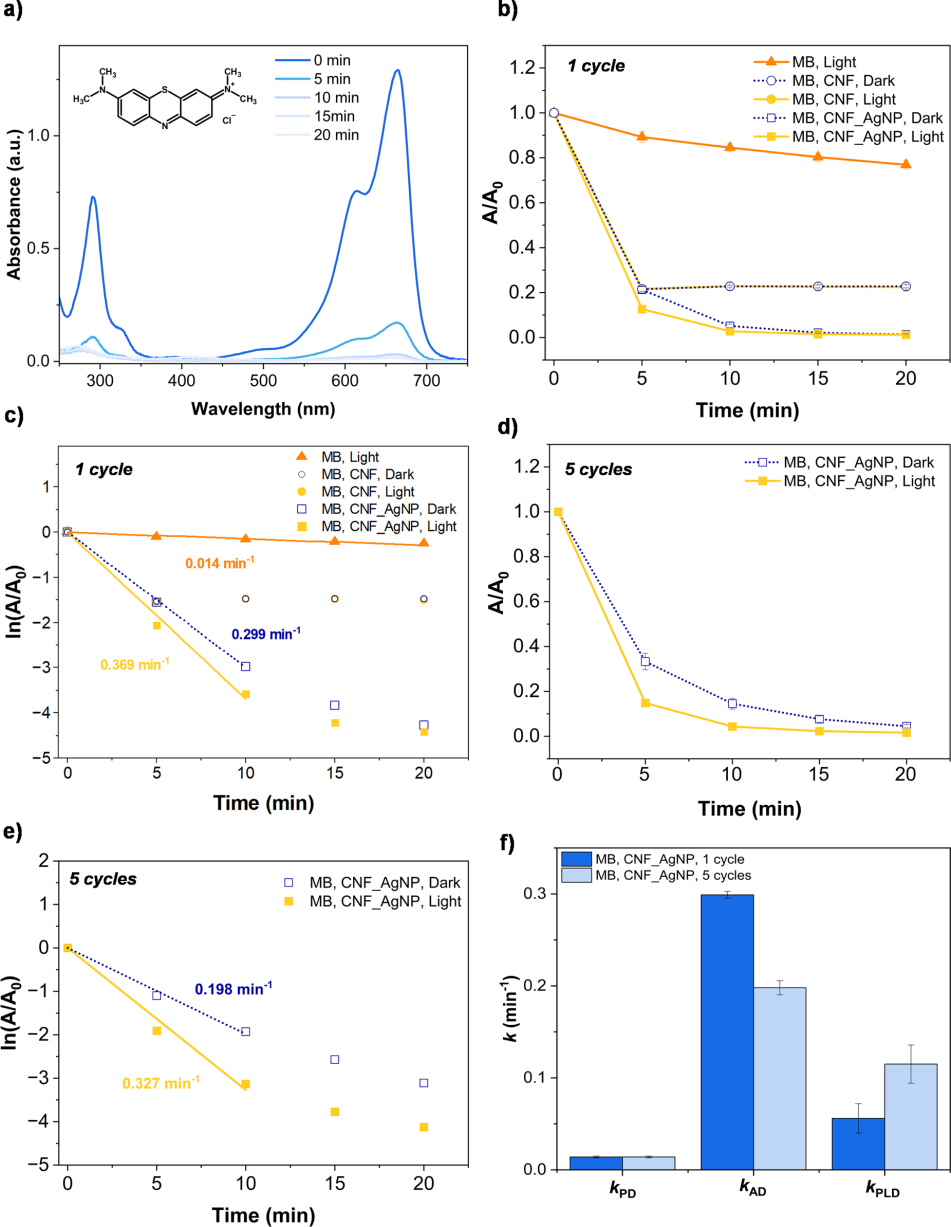
Performance of CNF and CNF_AgNP membranes in MB removal from water. (a) UV/vis spectra showing MB removal efficiency over time using CNF_AgNP membranes under blue light illumination. (b) MB removal kinetics (*A*/*A*_0_ vs time) under different conditions: photodegradation (MB without membranes under blue light), adsorption in dark conditions (CNF and CNF_AgNP), and combined adsorption and plasmonic degradation under blue light (CNF_AgNP). (c) Pseudo-first order kinetics (ln­(*A*/*A*_0_) vs time) for MB removal reaction under the same conditions. Data represent averages of three independent measurements with three different membranes. Some error bars are smaller than the data points. (d,e) Reusability of CNF_AgNP membranes: average of two sets of five subsequent cycles of MB degradation experiments under blue light and dark conditions, with (d) showing *A*/*A*_0_ vs time and (e) the corresponding pseudo-first-order kinetics plots (ln­(*A*/*A*_0_) vs time). The first spectrum (“0 min”) corresponds to the untreated MB solution. (f) Comparative analysis of reaction rate constants for photodegradation (*k*_PD_), adsorption (*k*_AD_) and plasmonic decomposition (*k*_PLD_) over multiple cycle experiments. Experimental conditions: [MB]_0_ = 0.02 mM; m­[CNF] = m­[CNF_AgNP] = (27.00 ± 0.13) mg, illumination power ≈ 0.5 W/cm^2^.

Results show that the photodegradation of MB by blue light alone without any material occurs, but with rather low yield (resulting in *A*/*A*_0_ = 0.77 ± 0.02 after 20 min), while the presence of both membranes (CNF and CNF_AgNP) significantly enhanced removal. CNF membranes, lacking AgNPs, removed ∼80% MB within 20 min by adsorption (*A*/*A*_0_ = 0.23 ± 0.01) purely due to nanocellulose’s well-known adsorptive properties,^52–54^ attributed to its functional groups that enhance molecular affinity at the CNF surface. The CNF membranes exhibited no photocatalytic activity, confirming that blue light illumination did not influence removal kinetics. In contrast, CNF_AgNP membranes achieved >90% removal of MB (*A*/*A*_0_ = 0.014 ± 0.001 in dark; *A*/*A*_0_ = 0.012 ± 0.001 under illumination after 20 min). It is interesting to note that the presence of AgNPs already significantly enhances the adsorption of MB even under dark conditions. This is attributed to the additional carboxyl groups present in the CNF_AgNP membranes as well as the enhanced surface area.

The kinetics are analyzed with a pseudo-first-order model (Figure 2c), with the total removal rate constant *k* incorporating contributions from photodegradation (*k*_PD_), adsorption (*k*_Ad_), and adsorption with simultaneous plasmonic degradation (*k*_PLD_)­

$$\frac{d\left[A\right]}{dt}=-k\cdot \left[A\right]=-\left(k_{PD}+k_{Ad}+k_{PLD}\right)\cdot \left[A\right]$$

here, [*A*] denotes the concentration of the organic pollutant (e.g., MB or Van) at time *t*. According to the results shown in Figure 2c, the determined photodegradation rate constant (*k*_PD_) is (0.014 ± 0.001) min^–1^, confirming the limited effectiveness of blue light alone. The adsorption rate constant (*k*_AD_) is determined to be (0.299 ± 0.004) min^–1^ (the rate constant for MB removal under dark conditions), indicating that adsorption is the fastest contributor for MB removal from solution. The rate constant for MB removal under light conditions is (0.369 ± 0.016) min^–1^, giving rise to a plasmonic degradation rate constant (*k*_PLD_) of (0.057 ± 0.001) min^1^. This value is considerably larger than the photodegradation rate constant (*k*_PD_), and it should be emphasized that this value accounts only for the additional removal of MB from solution due to plasmonic degradation at the plasmonic membrane and does not directly reflect the direct plasmon-induced decomposition reaction.

In order to differentiate more clearly between adsorption under dark conditions and plasmonic degradation, we attempted to determine the rate of MB removal with a CNF_AgNP membrane fully loaded with MB. However, the adsorption capacity of CNF_AgNP is so high that very large MB concentrations are required, which then leads to MB desorption in a fresh solution to an extent that prevents an accurate determination of MB removal upon irradiation. Instead, repeated cycle experiments were conducted in which the same membrane sample that was used for MB removal was subjected to a fresh solution of MB. Figure 2d compares MB degradation under blue light using CNF_AgNP membranes with adsorption-only membranes (dark conditions) over five cycles (average rate constant values calculated from two independent experiment series comprising five cycles). Now the difference between dark and light conditions becomes more pronounced because the adsorption process slows down by increasing the number of cycles (because the adsorption capacity reduces with every cycle), while the plasmonic degradation can be enhanced due to a presumably larger concentration of MB close to the nanoparticles. Figure 2e shows the corresponding pseudo-first-order kinetics. The overall reaction rate for CNF_AgNP membranes in the first cycle was (0.369 ± 0.016) min^–1^, while the average of five cycles slightly decreased to (0.327 ± 0.019) min^–1^, presumably due to slower adsorption. This is indicated by a comparative analysis of these rate constants, as shown in Figure 2f. Adsorption efficiency appears to decline in repeated cycles, while *k*_PLD_ increases from (0.057 ± 0.001) min^–1^ in the first cycle to (0.115 ± 0.021) min^–1^ averaged over five cycles. This suggests that the plasmonic effect remains stable and may even be enhanced over time, likely due to the more efficient exploitation of plasmonically active sites, which partially compensates for the reduced adsorption. These findings indicate that the AgNPs facilitate continuous electron transfer and localized heat generation, thereby sustaining the degradation process over time and demonstrating that CNF_AgNP membranes remain effective upon reuse.

No AgNP leaching was observed during the chemical reaction experiments, even under higher stirring speeds, as no absorbance peak at 400 nm was observed in the UV–vis spectra (Figure S5). This indicates a strong attachment of the AgNPs to the CNF matrix, validating their stability and highlighting the potential of these membranes for water treatment applications without AgNP release.^55^

The plasmonic degradation of MB has been studied previously under different conditions; nevertheless, the detailed degradation mechanism still remains elusive.^56^ On Au nanoparticles upon irradiation with 633 nm light, MB decomposes by demethylation, forming different forms of Azure and eventually resulting in the formation of thionine.^57^ This is enabled by the plasmonic E-field enhancement, leading to effective photoexcitation of the S_0_ → S_1_ transition in MB. The photodecomposition under these conditions is facilitated by the formation of singlet oxygen. In a plasmonic gold nanorod–MB system, visible-light excitation of the LSPR was shown to induce plasmon-induced resonance energy transfer, electronically exciting MB molecules and triggering their photo-oxidative polymerization, as evidenced by single-particle spectroelectrochemical analysis and theoretical modeling that excluded hot-electron and purely thermal pathways. A similar plasmon-mediated energy-transfer mechanism may be operative in our CNF–AgNP membrane under irradiation.^58^ On AgNPs, it was demonstrated that at 785 nm, a direct electron transfer to MB is possible (chemical induced damping, CID), resulting in photodesorption and photodecomposition.^59^ However, plasmonic degradation on AgNPs using blue light, as in the current work, is barely explored and needs detailed future studies.

#### 3.2.2 Van Removal: Adsorption Mechanism and Plasmon-Assisted Degradation

A separate set of experiments was conducted to evaluate the removal of Van, an antibiotic of great environmental concern due to its persistence in wastewater^60,61^ and role in antibiotic resistance in bacteria.^62^

Figure 3a shows the UV–vis spectra of Van solutions treated with CNF_AgNP membranes under blue light, demonstrating a progressive decrease in the absorbance at 280 nm. Unlike MB, Van did not undergo significant photodegradation under blue light alone, confirming its stability under these conditions. Figure 3b presents the removal kinetics of Van (*A*/*A*_0_ vs time) across different experimental conditions, revealing different, distinct adsorption behaviors compared to MB. While CNF membranes removed only ∼20% of Van after 20 min (dark and light: *A*/*A*_0_ = 0.82 ± 0.02), CNF_AgNP membranes exhibited much higher efficiency (∼93% removal, *A*/*A*_0_ = 0.07 ± 0.01), even in the absence of illumination. The enhanced adsorption observed in CNF_AgNP can arise from the strong interactions between Van and AgNPs, likely facilitated by the nanoparticle’s high surface area and the increased negative charge from carboxyl groups, as confirmed previously by FTIR analysis (Figure S2).

**Figure 3:**
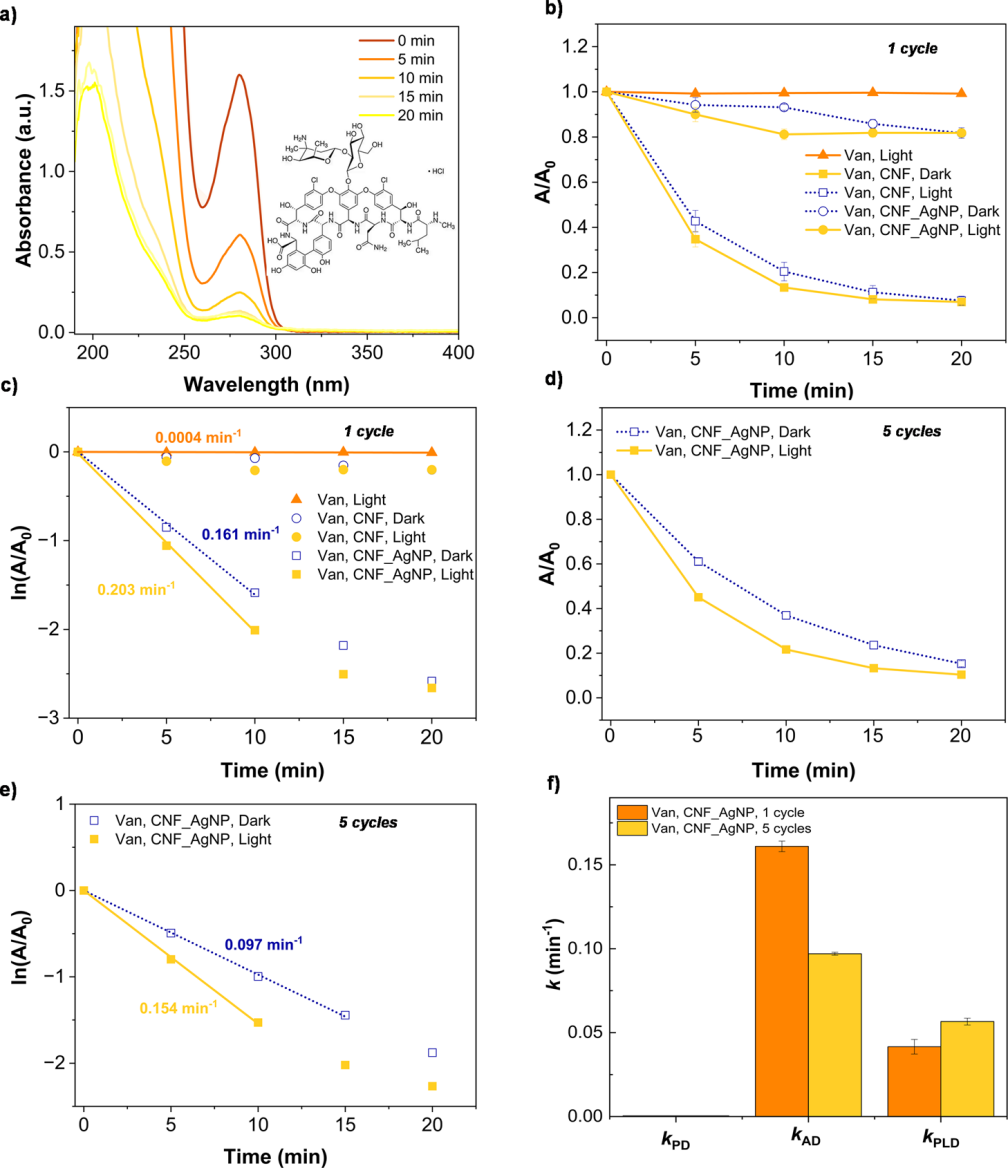
Performance of CNF and CNF_AgNP membranes in Van removal from water. (a) UV–vis spectra showing Van removal efficiency over time using CNF_AgNP under blue light illumination. (b) Van removal kinetics (*A*/*A*_0_ vs time) under different conditions: photodegradation (Van without membranes under blue light), adsorption in dark conditions (CNF, CNF_AgNP), and combined adsorption and plasmonic degradation under blue light (CNF_AgNP). (c) Pseudo-first-order kinetics (ln­(*A*/*A*_0_) vs time) for Van removal under the same conditions. Some error bars are smaller than the data points. (d,e) Reusability of CNF_AgNP membranes: average of two sets of five subsequent cycles of Van removal experiments under blue light and dark conditions, with (d) showing *A*/*A*_0_ vs time and (e) the corresponding pseudo-first-order kinetics plots (ln­(*A*/*A*_0_) vs time). The first spectrum (“0 min”) corresponds to the untreated Van solution. (f) Comparative analysis of reaction rate constants for photodegradation (*k*_PD_), adsorption (*k*_AD_), and plasmonic decomposition (*k*_PLD_) over multiple cycles. Experimental conditions: [vancomycin]_0_ = 0.25 mM; m­[CNF] = m­[CNF_AgNP] = 27.00 ± 0.13 mg; illumination power ≈ 0.5 W/cm^2^.

The kinetics analysis based on pseudo-first-order kinetics (Figure 3c) shows a higher rate constant for irradiated CNF_AgNP membranes compared to nonirradiated CNF_AgNP membranes, suggesting an additional plasmon-assisted degradation mechanism beyond adsorption. This effect is supported by Figure 3d, which compares Van removal across five subsequent cycles under light and dark conditions, with Figure 3e showing the corresponding pseudo-first-order kinetics. Following the approach used in the MB studies, the adsorption rate constant for Van (*k*_AD_) was (0.161 ± 0.003) min^–1^ for the first cycle, decreasing to (0.097 ± 0.001) min^–1^ when averaged over five cycles. The photodegradation rate constant (*k*_PD_) is very low at 0.0004 min^–1^, confirming that blue light alone is insufficient for Van breakdown. However, the plasmonic degradation rate constant (*k*_PLD_) increased from (0.042 ± 0.004) min^–1^ in the first cycle to (0.057 ± 0.002) min^–1^ when averaged over five cycles, indicating an effective plasmonic decomposition. Figure 3f compares these rate constants, highlighting the transition from adsorption-dominated removal (high *k*_AD_) to plasmonic-assisted degradation (higher *k*_PLD_) over repeated cycles. The overall reaction rate for CNF_AgNP membranes in the first cycle was (0.203 ± 0.003) min^–1^, decreasing slightly to (0.154 ± 0.002) min^–1^ in later cycles. Similar to MB removal, the increase in *k*_PLD_ despite the reduction in *k*_AD_ suggests that the CNF_AgNP membranes remain effective upon reuse. Those results agree with previous work on the use of titanium dioxide nanostructures combined with plasmonic gold nanoparticles, which enhance the photocatalytic degradation of Van.^63^ It was suggested that Van is decomposed by electron transfer, and that the improvement of the photocatalytic performance occurs through different mechanisms: increased light absorption by Au in the visible spectrum, enhanced electron–hole pair generation, and improved charge separation and transfer.^63^

Likewise, the effect of different wavelengths on the removal of Van was tested. Figure 4a presents the results of the natural-logarithm transformation ln­(*A*/*A*_0_) based on the average of the Van removal kinetics (*A*/*A*_0_ vs time) presented in Supporting Information (Figure S6). The determination of the pseudo-first-order rate constant k (min^–1^) for each experimental condition monitored at 280 nm is presented. Accordingly, the irradiation of blue light (457 nm) leads to the strongest enhancement of Van removal (*k* = 0.104 min^–1^) compared to dark conditions (*k* = 0.065 min^–1^), where only adsorption contributes to Van removal. Irradiation with green (523 nm), red light (623 nm), and white light led to the rate constants of *k* = 0.102 min^–1^, *k* = 0.90 min^–1,^ and *k* = 0.085 min^–1^, which are still significantly higher than under dark conditions. The drop in the removal rate at larger wavelengths is to be expected as the maximum of LSPR of AgNPs is at 380–450 nm. Nevertheless, the AgNPs in our plasmonic membrane are arranged at close distance, leading to a coupling of the LSPR and corresponding shifts and new features at higher wavelengths. This can be seen in the absorbance of the plasmonic CNF_AgNP membrane (Figure 4b), which shows a strong and almost constant absorption between 300 and 550 nm, followed by a drop in the red and near-infrared regime. These spectral features align well with the observation that both blue and green light give rise to very similar removal rate constants, while the one obtained by right light is slightly lower. When the LSPR are excited, enhanced electromagnetic fields arise around the nanoparticles, and nonthermal charge carriers are formed, followed by a temperature increase. All of these effects might contribute to a decomposition of Van, and the specific contributions and decomposition pathways need to be studied in follow-up experiments. A significant effect of heating is supported by the measurable temperature increase observed when the CNF_AgNP membrane is irradiated with light, indicating heat generation associated with plasmonic excitation.

**Figure 4:**
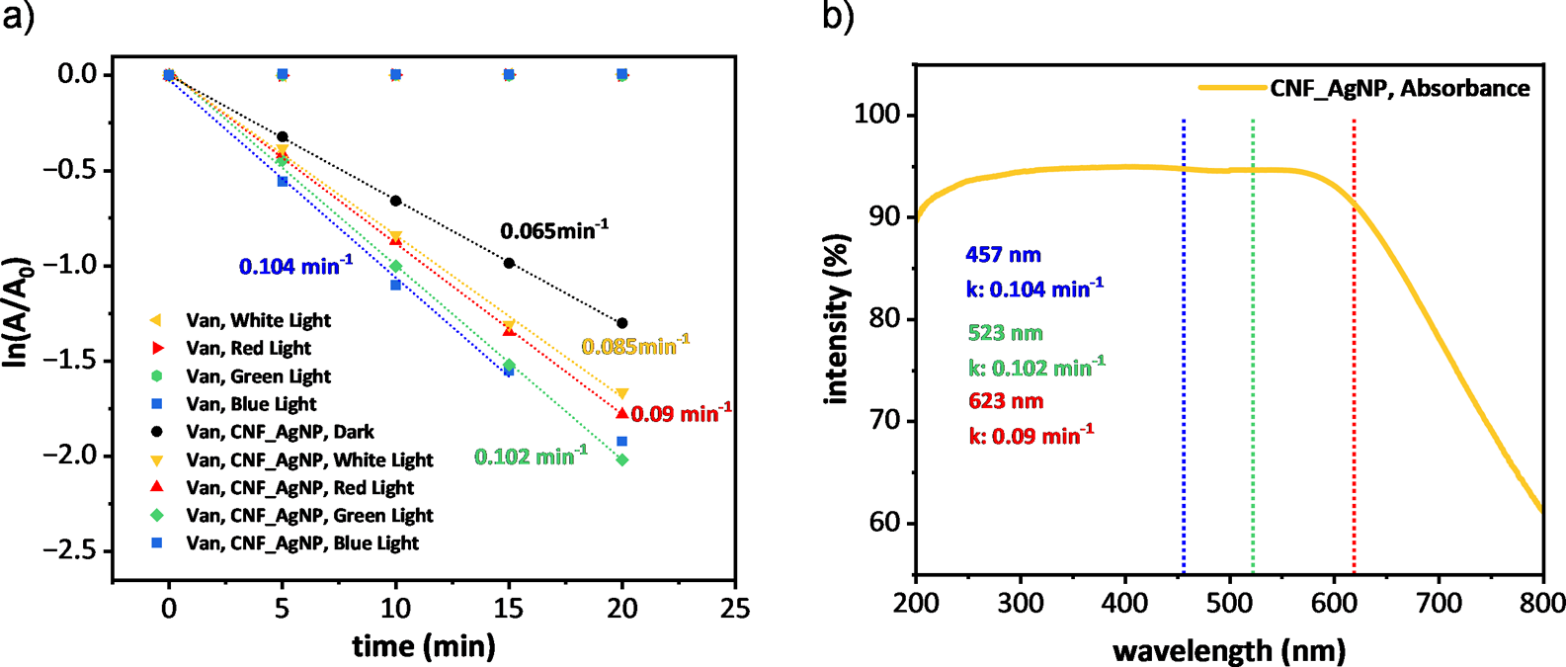
Van removal with the CNF_AgNP membrane and, as a control, without any material, under different illumination conditions: green light, blue light, red light, white light, and dark conditions (no irradiation). (a) Determination of the pseudo-first-order rate constant k (min^–1^) for each experimental condition monitored at 280 nm. (b) Absorbance spectrum of the dried CNF_AgNP membrane.

The value of the adsorption capacity (*q*_e_) of the CNF_AgNP for Van was determined to be 744 ± 42 mg·g^–1^. Compared to other systems applied for Van removal, this represents a remarkably high capacity: ionic-liquid modified activated carbon^64^ has an adsorption capacity of 132 mg·g^–1^, while powder activated carbon/magnetite composite (Fe_3_O_4_)^65^ has a capacity of 100 mg·g^–1^. Also, neutron-macroporous HA380 hemoperfusion resin basis^66^ was tested, and the Van capacity was determined to be 244 mg·g^–1^. This strong adsorption capacity is attributed to the high surface area of the composite nanocellulose–silver nanoparticle system, as well as to the high affinity of Van to AgNPs.

We hypothesized that plasmonic CNF_AgNP membranes irradiated with blue light could deactivate Van’s antibacterial properties. To test this, we assessed the in vitro susceptibility of *B. subtilis* to Van solutions treated with plasmonic CNF_AgNP membranes under irradiation.

Briefly, Van solutions were exposed to plasmonic CNF_AgNP membranes under blue light for 30 or 60 min and subsequently incubated with *B. subtilis*. Bacterial growth was monitored using an inhibition assay by measuring the optical density at 600 nm (OD_600_) at two time points: immediately after inoculation (0 h) and after 24 h of incubation.

Figure 5 presents a comparison of the bacterial growth under different experimental conditions. Control groups behaved as expected: untreated bacteria (no Van) showed significant growth over 24 h, as reflected by higher OD_600_ values, while the medium-only control remained stable, confirming the reliability of the assay. In the case of fresh Van solution (2 μg/mL), OD_600_ values remained low, confirming the antibiotic’s effectiveness in completely inhibiting bacterial growth. However, Van solutions treated with plasmonic CNF_AgNP membranes exhibited a significant increase in OD_600_ after 24 h of incubation, indicating bacterial growth. This result closely aligns with the untreated bacteria control, supporting the conclusion that the treatment with the plasmonic membrane effectively deactivated Van’s antibacterial function. The breakdown promoted by the plasmonic reaction leads to less harmful molecular byproducts, as previously demonstrated using Au-TiO_2_ nanoarrays and nanowires.^63,67^

**Figure 5:**
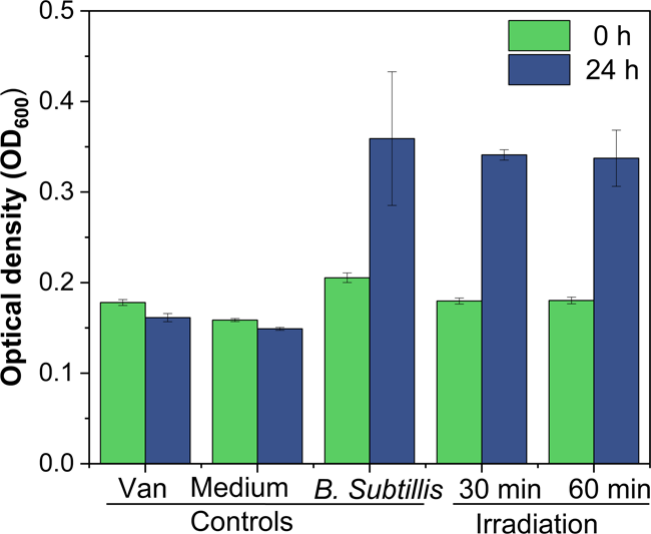
OD_600_ readings for the bacterial inhibition test of 2 μg/mL Van solutions treated with plasmonic CNF_AgNP membranes under blue light irradiation for 30 or 60 min, followed by incubation with *B. subtilis*. OD_600_ values were measured at 0 h (immediate reading) and 24 h (postincubation to assess bacterial growth). Control groups include Van solution without irradiation in the presence of bacteria (Van), medium, and pure bacteria (*B. subtilis*). Error bars represent ± standard deviation of triplicate experiments. All OD_600_ values are provided in the Supporting Information (Table S1).

### 3.3 Plasmonic Activity of CNF_AgNP Membranes

To further investigate the role of plasmonic activity in pollutant degradation, we examined the photothermal effect of CNF_AgNP membranes under prolonged blue light irradiation. This effect is crucial, as plasmonic excitation of AgNPs leads to localized heating and electron transfer, both of which can facilitate molecular dissociation.

To evaluate this plasmonic contribution, temperature measurements were recorded under three conditions: (i) water alone, (ii) water with a CNF membrane, and (iii) water with a CNF_AgNP membrane (Figure 6a). The presence of CNF_AgNP membranes resulted in a significant temperature increase, reaching an average of (62.1 ± 5.9) °C, whereas CNF membranes and water alone exhibited negligible temperature changes. This pronounced heating effect is a strong indicator of plasmonic activity and further supports the role of AgNPs in facilitating degradation through both photothermal and photocatalytic processes. These results align with our degradation studies using MB and Van, which independently confirm that the CNF_AgNP membrane enhances the breakdown of pollutants under irradiation.

**Figure 6:**
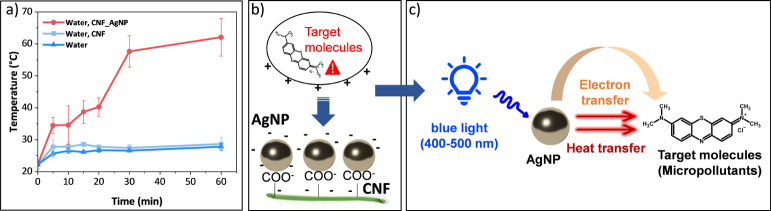
(a) Temperature measured inside the solution upon irradiation in the photoreactor: (i) water with a CNF_AgNP membrane, (ii) water with a CNF membrane, and (iii) water alone. (b) Representative schematic illustrating plasmonic reactions considering the charge effect on the CNF surface. (c) Overall mechanism of plasmonic degradation of pollutant molecules.

Figure 6b,c presents a schematic of this plasmonic reaction mechanism. Initially, CNF and AgNPs promote adsorption or electrostatic attraction, drawing pollutant molecules close to the AgNP surface (Figure 6b). Upon blue light excitation, the excitation of the LSPR of AgNPs leads to the release of electrons and generates localized heat, which can induce molecular dissociation (Figure 6c). Consequently, there is a pronounced synergistic effect when the two processes adsorption and plasmonic degradation are combined. The details of the plasmonic degradation of MB and Van under the conditions applied in the present work will be studied in further work.

## 4 Conclusion

This study demonstrates the potential of CNF_AgNP membranes as a multifunctional platform for the efficient removal of organic pollutants from water, using the synergistic mechanisms of plasmonic photocatalysis and adsorption, without the need for oxidative agents. The in situ generation of AgNPs within a CNF matrix enables effective removal of MB and Van under blue light irradiation, maintaining consistent performance over many cycles. The CNF matrix plays a dual role by stabilizing the AgNPs (eliminating the need for additional capping agents) and enhancing pollutant adsorption, which facilitates proximity-driven plasmonic catalytic activity, while preventing AgNP leaching. This dual functionality addresses critical challenges, such as nanoparticle aggregation, reusability, and secondary waste generation. It is important to highlight the advantages of plasmonic degradation reactions, which enable more efficient utilization of visible light, which makes plasmonic photocatalysis a promising approach for solar-driven antibiotic degradation, facilitating the breakdown of pollutants into less harmful molecules. This suggests that CNF_AgNP may be suitable for removing further emerging pollutants such as PFAS,^48^ pesticides, and pharmaceuticals.

These findings position CNF_AgNP membranes as a promising, scalable, and sustainable solution for plasmon-mediated water treatment under mild conditions, creating a catalytic material in which the pollutants are not only collected but also degraded, with the vision to create a self-cleaning filter material. Future work should focus on expanding the range of pollutants addressed by CNF_AgNP membranes and optimizing the membrane design for large-scale, real-world applications. Overall, our study advances the development of nanophotonics-based solutions in environmental remediation, contributing to global efforts to mitigate water pollution.

## Supplementary Material



## Data Availability

The data that support the findings of this study are openly available in Zenodo at https://doi.org/10.5281/zenodo.18074582.

## Notes

HR-TEM images of AgNPs, AFM image of the CNF–AgNP membrane, the rheological behavior of pure CNF and CNF_AgNP membranes, the storage modulus *G*′ and loss modulus *G*″ and the complex viscosity, FTIR and TGA measurements of CNF and CNF_AgNP membranes, UV–vis spectroscopy of the AgNP removed from CNF_AgNP membrane, the kinetics of the average of *A*/*A*_0_ measured for Van through the time under different wavelengths irradiation with and without CNF_AgNP membranes, and the measurements of *B. subtilis* growth in Van solutions after treatment with plasmonic CNF_AgNP membranes under blue light illumination (PDF)
