# Supplementary material for: Nanocellulose Membranes for Plasmon-Enhanced Removal of Organic Pollutants from Water
Source: ACS Appl Nano Mater. 2026 Jan 3;9(2):1129–41. doi: 10.1021/acsanm.5c04857 (PMC12813976; doi:10.1021/acsanm.5c04857)
Supplement: Supplementary file 1 [file an5c04857_si_001.pdf]

Supporting Information

Nanocellulose Membranes for Plasmon-Enhanced Removal of Organic Pollutants from Water

Sivoney Ferreira de Souza,[1], Christina Beresowski[1], Sabine Kosmella[1], João Ameixa[1,2], Bhanu Kiran Pothineni[3], Adrian Keller[3], Matthias Hartlieb[1,4], Andreas Taubert[1], Ilko Bald[1*]*

[1]Institute of Chemistry, University of Potsdam, Karl-Liebknecht-Str. 24-25, 14476, Potsdam, Germany
[2]Centre of Physics and Technological Research (CEFITEC), Department of Physics, NOVA School of Science and Technology, University NOVA of Lisbon, Campus de Caparica, 2829-516, Portugal
[3]Paderborn University, Technical and Macromolecular Chemistry, Warburger Str. 100, 33098, Paderborn, Germany
[4]Fraunhofer Institute for Applied Polymer Research (IAP), Geiselbergstraße 69, 14476, Potsdam, Germany

*Correspondence: ferreiradesouza@uni-potsdam.de, bald@uni-potsdam.de.

1. Materials and Methods

Materials

Reagents such as sodium hydroxide (NaOH), ammonium solution (NH_4OH), silver nitrate ($AgNO_3$), glycerol, methylene blue and vancomycin hydrochloride were purchased from Sigma-Aldrich. Cellulose nanofibers (CNF) were obtained from Nanografi (width: 10-20 nm, length 2-3 µm). *Bacillus subtilis* line DSM 5545 and LB Broth medium from Roth, Germany.

2. Results

Morphological Characterization

The high resolution-transmission electron microscopy (HR-TEM) images presented in **Figure S3** reveal in detail the morphology and shapes of the AgNP synthesized on CNF-AgNP membrane. Also the diameters of the AgNP images were measured using ImageJ 1.54g software.

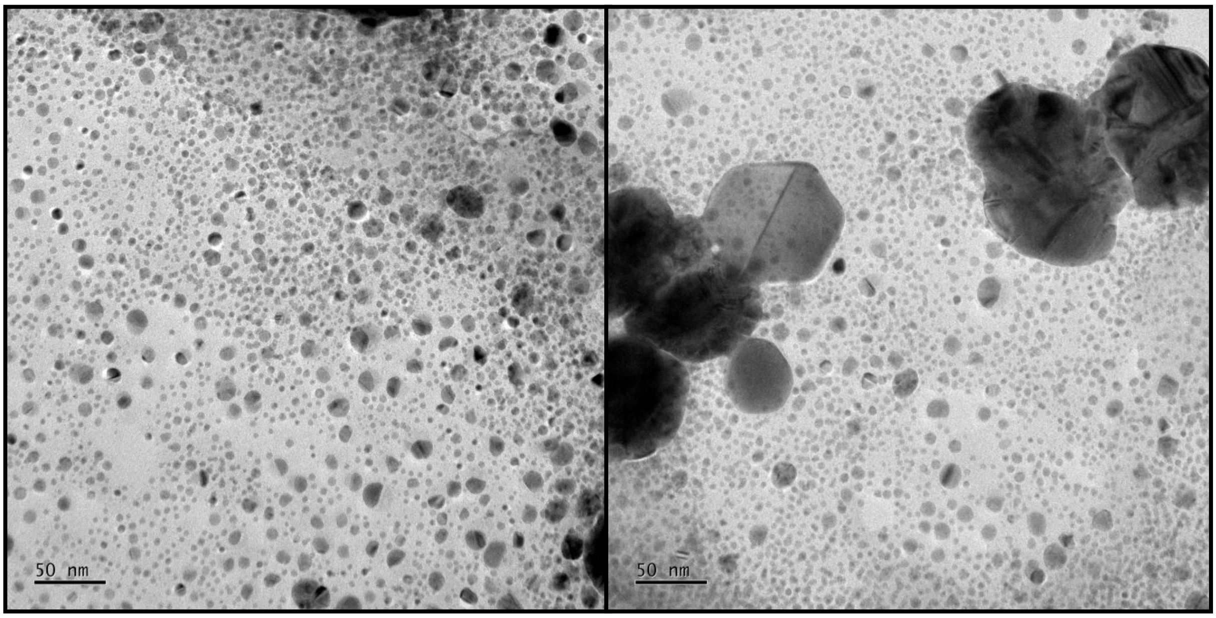

Figure S1. HR-TEM images of AgNPs that were used to measure the size distribution using Image J software.

Morphological characterization of CNF_AgNP membrane

AFM (atomic force microscopy) images of the CNF–AgNP membrane was performed. These AFM images clearly show the organization of the CNF network within the composite.

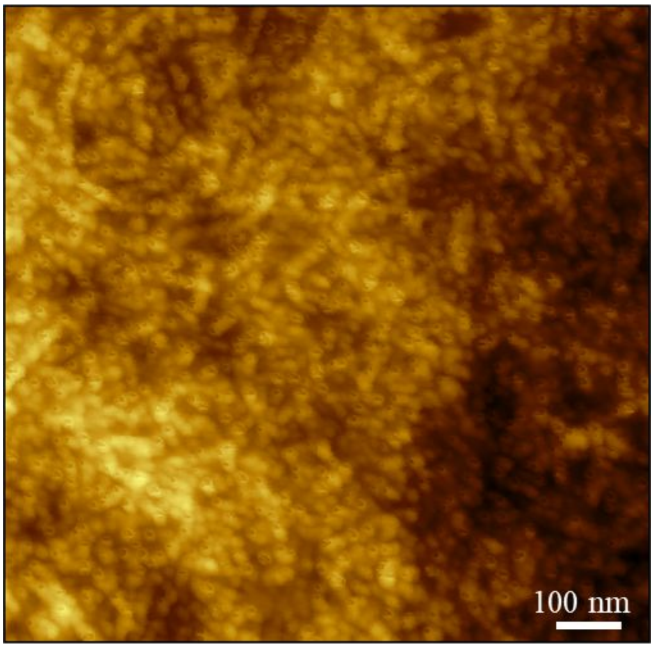

Figure S2. AFM image of the CNF-AgNP membrane acquired using tapping mode. The image treatment was done in Gwyddion. The image represents an area of 1 x 1 μm^2.

Rheological behavior of pure CNF and CNF_AgNP membranes

The entangled and interlocking nanofiber network enhances the membrane's stability, even in aqueous environments. The *in situ* incorporation of AgNPs preserves the flexibility of the CNF membranes while maintaining their structural robustness, as the nanoparticles are tightly integrated within the matrix rather than loosely deposited on the surface.

The mechanical properties of CNF_AgNP membranes (Figure S1) were evaluated using a rheometer with a plate-to-plate geometry. Both pure CNF and CNF_AgNP membranes exhibit solid-like behavior, with the storage modulus (G') consistently higher than the loss modulus (G"), suggesting a predominantly elastic response. The storage modulus increases with frequency, reflecting the reinforcement and organization of the CNF network, likely due to the high aspect ratio of CNFs, which enhances yield stress and promotes entanglement[1] .

Complex viscosity measurements further confirm minimal interference from AgNPs. Both pure CNF and dCNF_AgNP membranes exhibit similar viscosity trends, with a decrease in viscosity at higher frequencies as nanoparticle mobility is restricted within the stiffened CNF matrix. This aligns with the observed increase in the elastic modulus (G′), highlighting the membranes' mechanical robustness and their potential for water treatment applications.

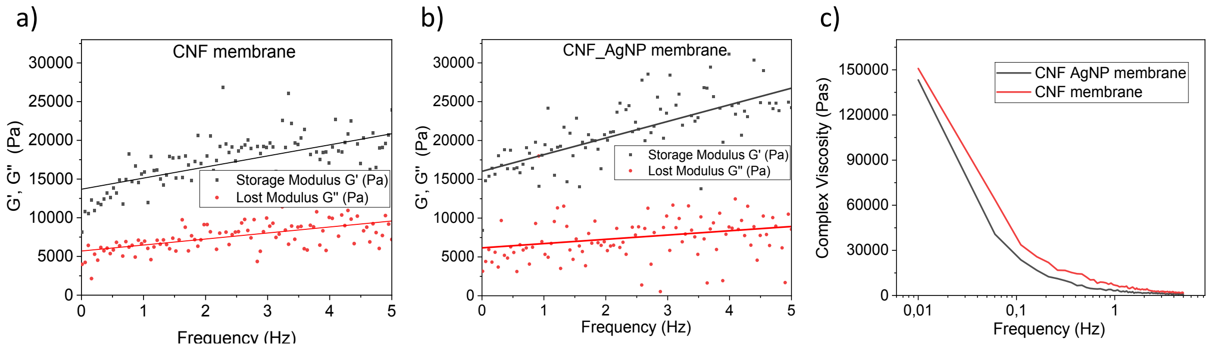

Figure S3. Rheological behavior of pure CNF and CNF_AgNP membranes, the storage modulus G′ (black line) and loss modulus G″ (red line): (a) Pure CNF and (b) CNF_AgNP membranes. (c) Complex viscosity (η∗) obtained from oscillatory shear measurements for pure CNF (red) and CNF_AgNP membranes (black).

Chemical and thermal characterization of the membranes

Fourier-transform IR (FTIR) spectroscopy was used to analyze the chemical composition of pure CNF and CNF_AgNP membranes (**Figure S4a**). Both samples displayed characteristic peaks approximately 3310 cm^{-1}, 2880 cm^{-1}, and 1035 cm^{-1}, corresponding to OH, CH_2, and CO vibrations, respectively. Notably, CNF_AgNP membrane exhibited additional peaks at 1735 cm^{-1}, 1375 cm^{-1}, and 1235 cm^{-1}. The peak at 1735 cm^{-1} corresponds to CO vibrations, while the

peak at 1375 cm^{-1} is associated with carboxylate (COO-) groups, suggesting the introduction of negative charges. The peak at 1235 cm^{-1} is attributed to CO vibrations from sugar units in the cellulose matrix. These additional peaks suggest oxidation of the cellulose, which likely acted as a reducing agent during the *in situ* synthesis of AgNPs.

Thermogravimetric analysis (TGA) further highlighted the thermal stability of the membranes (**Figure S4b**). While both CNF and CNF_AgNP membranes demonstrated a two-step degradation pattern, the CNF_AgNP membrane exhibited enhanced thermal stability compared to pure CNF. This improvement is likely due to the presence of AgNPs, which not only contribute to photocatalytic functionality but also can restrict thermal degradation pathways by stabilizing the cellulose structure. The increased thermal stability supports the potential of CNF_AgNP membranes for robust application.

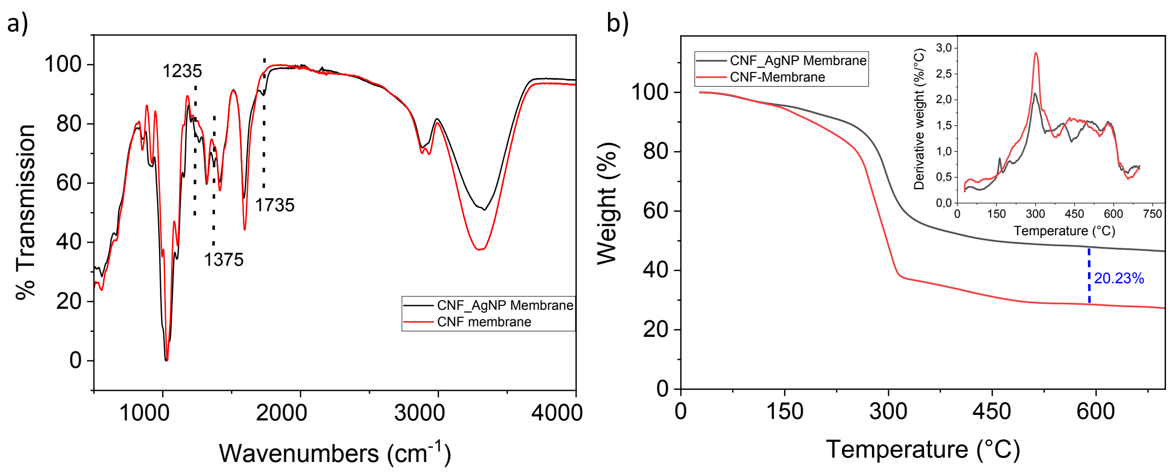

Figure S4. Chemical and thermal characterization of CNF and CNF_AgNP membranes. (a) FTIR spectra of pure CNF (blue) and CNF_AgNP (red), highlighting functional group changes due to AgNP incorporation. (b) Thermogravimetric analysis (TGA) curves of pure CNF (black) and CNF_AgNP (red), demonstrating improved thermal stability with AgNP integration.

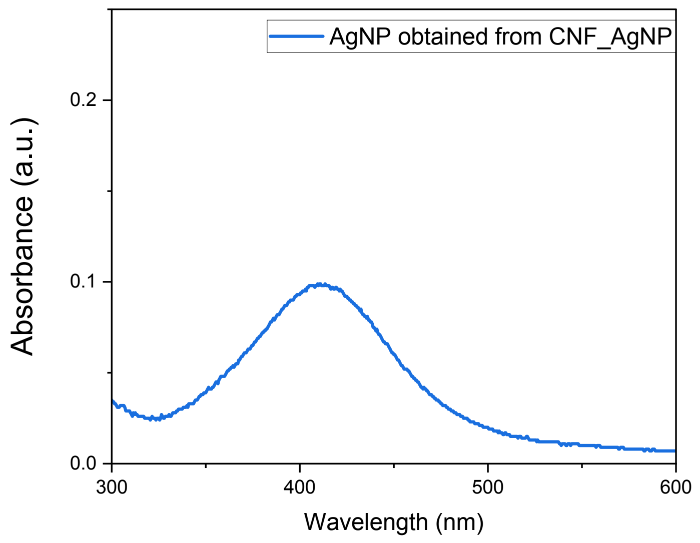

Figure S5. UV-Vis spectroscopy of the CNF_AgNP after breaking down into small nanoparticles and after sonication as TEM and TEM-HR sample preparations.

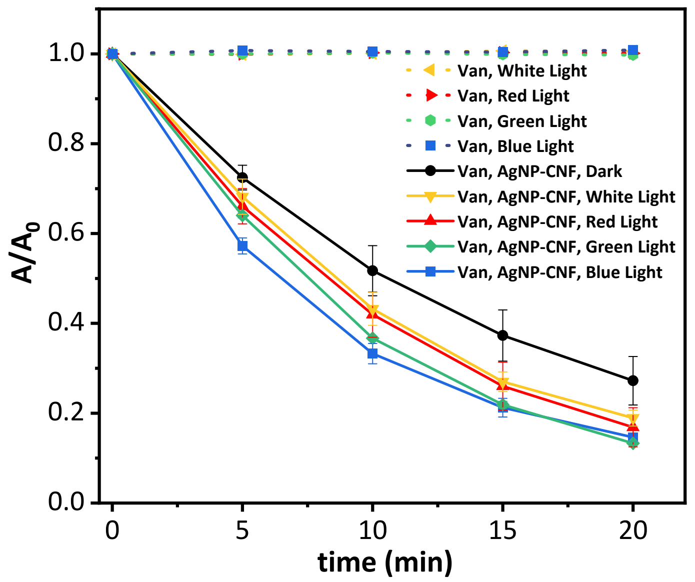

Figure S6. Kinetics of the average of A/A_0 measured through the time following the peak of 280 nm under different wavelengths irradiation: Van without membranes under irradiation; dark conditions (adsorption of Van solution on the CNF_AgNP membrane) and the plasmonic reaction with the different wavelength.

Bacterial inhibition studies

Table S1. Optical density (OD_{600}) measurements of *Bacillus subtilis* growth in vancomycin solutions treated with plasmonic CNF_AgNP membranes under blue light illumination. Values represent the mean ± standard deviation (SD) from triplicate experiments.

		OD_{600} ± SD	
Vancomycin	Condition	0 h	24 h
2 µg/mL	No exposure	0.178 ± 0.003	0.161 ± 0.005
	30 min treatment	0.180 ± 0.003	0.341 ± 0.006
	60 min treatment	0.180 ± 0.004	0.341 ± 0.006
	B. subtilis control	0.205 ± 0.005	0.359 ± 0.074
	LB medium control	0.159 ± 0.002	0.149 ± 0.002
3 µg/mL	No exposure	0.164 ± 0.004	0.142 ± 0.002
	30 min treatment	0.162 ± 0.001	0.297 ± 0.004
	60 min treatment	0.175 ± 0.003	0.34 ± 0.01
	B. subtilis control	0.205 ± 0.005	0.359 ± 0.074
	LB medium control	0.159 ± 0.002	0.149 ± 0.002

References

(1) Im, W.; Park, S. Y.; Goo, S.; Yook, S.; Lee, H. L.; Yang, G.; Youn, H. J. Incorporation of CNF with Different Charge Property into PVP Hydrogel and Its Characteristics. *Nanomaterials* **2021**, *11* (2). DOI: 10.3390/nano11020426. Published Online: Feb. 8, 2021.
